# Preclinical Safety Evaluation of Intranasally Delivered Human Mesenchymal Stem Cells in Juvenile Mice

**DOI:** 10.3390/cancers13051169

**Published:** 2021-03-09

**Authors:** Yolanda Aguilera, Nuria Mellado-Damas, Laura Olmedo-Moreno, Víctor López, Concepción Panadero-Morón, Marina Benito, Hugo Guerrero-Cázares, Catalina Márquez-Vega, Alejandro Martín-Montalvo, Vivian Capilla-González

**Affiliations:** 1 Andalusian Molecular Biology and Regenerative Medicine Centre (CABIMER)-CSIC-US-UPO, Department of Regeneration and Cell Therapy, 41092 Seville, Spain; yolanda.aguilera@cabimer.es (Y.A.); nuria.mellado@cabimer.es (N.M.-D.); laura.olmedo@cabimer.es (L.O.-M.); victorld1991@gmail.com (V.L.); conchipm98@gmail.com (C.P.-M.); alejandro.martinmontalvo@cabimer.es (A.M.-M.); 2 Research Magnetic Resonance Unit, Hospital Nacional de Parapléjicos, 45004 Toledo, Spain; mbenitov@sescam.jccm.es; 3 Department of Neurosurgery, Mayo Clinic, Jacksonville, FL 32224, USA; Guerrero-Cazares.Hugo@mayo.edu; 4 Pediatric Oncology Unit, Hospital Virgen del Rocio, 41013 Seville, Spain; catalina.marquez.sspa@juntadeandalucia.es

**Keywords:** mesenchymal stem cells, cell therapy, intranasal delivery, biosafety, nervous system disorders

## Abstract

**Simple Summary:**

The concept of utilizing mesenchymal stem cells for the treatment of central nervous system disorders has progressed from preclinical studies to clinical trials. While promising, the effectiveness of cell therapy is hampered by the route used to deliver cells into the brain. In this context, intranasal cell administration has boomed over the past few years as an effective cell delivery method. However, comprehensive safety studies are required before translation to the clinic. Our study shed light on how intranasally administrated mesenchymal stem cells may be used to safely treat neurological disorders.

**Abstract:**

Mesenchymal stem cell (MSC)-based therapy is a promising therapeutic approach in the management of several pathologies, including central nervous system diseases. Previously, we demonstrated the therapeutic potential of human adipose-derived MSCs for neurological sequelae of oncological radiotherapy using the intranasal route as a non-invasive delivery method. However, a comprehensive investigation of the safety of intranasal MSC treatment should be performed before clinical applications. Here, we cultured human MSCs in compliance with quality control standards and administrated repeated doses of cells into the nostrils of juvenile immunodeficient mice, mimicking the design of a subsequent clinical trial. Short- and long-term effects of cell administration were evaluated by in vivo and ex vivo studies. No serious adverse events were reported on mouse welfare, behavioral performances, and blood plasma analysis. Magnetic resonance study and histological analysis did not reveal tumor formation or other abnormalities in the examined organs of mice receiving MSCs. Biodistribution study reveals a progressive disappearance of transplanted cells that was further supported by an absent expression of human GAPDH gene in the major organs of transplanted mice. Our data indicate that the intranasal application of MSCs is a safe, simple and non-invasive strategy and encourage its use in future clinical trials.

## 1. Introduction

Cell therapy is an important component of regenerative medicine that has shown promising results over the past few years. Mesenchymal stem cells (MSCs) are one of the most extensively explored cell type in cell-based treatments due to numerous advantages [1,2,3,4,5,6,7,8]. Among them, MSCs possess multilineage differentiation potential, tropism towards damaged tissues, and paracrine properties that contribute to tissue repair and regeneration. Importantly, MSCs can be obtained from easily accessible sources in juvenile or adult individuals (e.g., adipose tissue, bone marrow and dental pulp), avoiding ethical issues associated with the use of fetal or embryonic tissue [9]. Furthermore, MSCs can be rapidly expanded in large-scale and used as clinical therapeutics, when they are produced in compliance with the Good Manufacturing Practice (GMP) [10].

MSC-based therapy has emerged as an attractive alternative in the treatment of neurological disorders, such as stroke, brain cancer, Friedreich’s ataxia, traumatic brain injury, and white matter damage, among others [11,12,13,14,15,16,17]. However, the route of cell delivery to the brain may represent a major limitation for the effectiveness of central nervous system (CNS) therapies. Systemic administration is a widely used method in preclinical and clinical research, but this route has a reduced therapeutic effectiveness because the blood–brain barrier (BBB) impedes sufficient entry of transplanted cells into the damaged brain area [18,19,20]. Another frequent method to deliver cells into the brain is the intracranial transplantation, which enables greater grafting of cells into the brain than systemic injections. Although effective, intracranial transplantation requires invasive procedures that compromise host safety [21]. These limitations have led researchers to seek less invasive, but effective methods to deliver cells for the treatment of neurological disorders. In this context, intranasal delivery is an alternative option to administrate cells into the brain, promoting efficient tissue regeneration [22]. In a previous study, we demonstrated that repeated intranasal applications of human MSCs were effective to prevent neurocognitive decline following radiotherapy, by limiting inflammation, neuronal loss and oxidative damage shortly after cell transplantation in mice [23]. These results hold promise in the prevention of neurological sequelae following radiotherapy in brain cancer patients, and more particularly in pediatric patients whose developing brains are more sensitive to radiation [24,25,26,27]. However, the safety of repeated intranasal delivery of MSCs needs to be deeply evaluated before translating this strategy into clinical application.

Here, we investigate the biosafety of multiple doses of intranasally administrated human MSCs in mice from early after-weaning to adult life. Exhaustive in vivo and ex vivo analyses were conducted to identify safety issues on the major organs, with special emphasis on the brain. This safety evaluation, together with our previous efficacy study [23], represents a step forward in the clinical use of repeated intranasal application of MSCs to treat the side effects of cancer treatments and other neurological disorders.

## 2. Materials and Methods

### 2.1. Production and Quality Control Standards for MSCs

Cryopreserved human adipose derived MSCs were used to produce the 4 cell doses employed for this study. Briefly, MSCs (ATCC, PCS-500-011™) were grown in Dulbecco’s Modified Eagle Medium (DMEM; Life Technologies, Carlsbad, CA, USA) supplemented with 10% fetal bovine sera and 1% penicillin-streptomycin, and incubated at 37 °C in a 20% O_2_ and 5% CO_2_ humidified atmosphere. Media were changed every 2–3 days. All cell doses (5·10^5^ of cells/dose) were prepared with low-passage MSCs (4–5 passages) that were resuspended in phosphate-buffered saline (PBS). Prior cell administration, quality control (QC) standards were performed according to GMP requirements (see Supporting Information for expanded material and methods).

### 2.2. Animals

Six-week-old male immunodeficient athymic nude mice (Charles River Laboratories, Barcelona, Spain) were randomly divided into four experimental groups: intact control mice (CTR group; *n* = 12), mice receiving intranasal PBS (PBS group; *n* = 20), mice receiving intranasal MSCs (MSC group; *n* = 20), and mice receiving intranasal positive control cancer cells (U87 group; *n* = 8). We decided to include the U87 group as a positive control group to demonstrate that transplanted cancer cells (i.e., U87, which is a human glioma cell line capable of inducing tumors in nude mice [28]) may induce adverse events, while MSCs do not. Mice were monitored for a short- and long-term study (12 and 24 weeks post-transplant, respectively). Analysis beyond 24 weeks post-transplant were not considered in this study to avoid spontaneous atypical masses in the nude mice. All animals were housed in a specific pathogen free animal facility on a 12-h light/dark cycle, with stable temperature (22 °C) and humidity (60%), and with food and water available ad libitum. All animal handling procedures were approved by the CABIMER Ethics Committee for Animal Experimentation, and complied with national and European Union legislation (Spanish RD 53/2013 and EU Directive 2010/63) for the protection of animals used for scientific purposes. All animal experiments were conducted under Good Laboratory Practices conditions.

### 2.3. Intranasal Cell Administration and Biodistribution

Animals were anesthetized and placed in a supine position to administrate total of 100 U of hyaluronidase as two repeated inoculations in each nostril with 5-min intervals (3 μL per inoculation). After 30 min, 5·10^5^ of cells (MSC or U87) were delivered as 2 repeated inoculations in each nostril with 5-min intervals (3 μL per inoculation). Mice received a dose of cells per week during 4 consecutive weeks. PBS mice received hyaluronidase followed by PBS. For evaluation of cell biodistribution, cultured MSCs and U87 cells were incubated with 400 μg/mL XenoLight DiR fluorescent dye (Perkin Elmer, Inc., Boston, MA, USA) for 30 min at 37 °C before transplantation. Transplanted mice were periodically imaged during the study using an IVIS Imaging System 200 Series (Caliper Life Science, Hopkinton, MA, USA).

### 2.4. Welfare Assessing

To assess animal welfare, we followed a previous published protocol that evaluates parameters corresponding to the 12 welfare criteria established by the Welfare Quality^®^ project [29]. These criteria regarded to good feeding, housing, health and appropriate behavior. Welfare assessment was carried out over the whole study period.

### 2.5. Behavioral Tests

Changes on neurological status and motor performance were evaluated at the short- and long-term after cell delivery (i.e., 12 weeks and 24 weeks post-transplantation, respectively) using a battery of behavioral tests, following previously described protocols. First, olfaction was evaluated by measuring odor discrimination capacity in a two-odorants test (habituation-dishabituation test) [30]. Second, cognition was assessed by performing the novel object recognition task with a long habituation phase, using odorless objects that do not retain any olfactory cues [31]. Third, muscle strength was evaluated by the wirehang test [32]. Finally, motor coordination was evaluated by rotarod performance [33].

### 2.6. Blood Sample Collection and Biochemical Determinations

Blood samples were collected in EDTA-containing microcentrifuge tubes from the tail vein of 12 h fasted mice, on week 12 and week 24 post cell delivery. Plasma was obtained by centrifugation (2000× *g*, 10 min) and used to determine the levels of biochemical metabolites and oxidative stress parameters, using commercially available reagents with the Cobas Integra 400 plus analyzer (Roche Diagnostic, Germany) or a spectrophotometer. The cellular components of blood were used when necessary.

### 2.7. Cytokines Bio-Plex Immunoassay

Plasma samples were assayed using the Bio-Plex Pro™ Mouse Cytokine Immunoassay kit (Bio-Rad Laboratories, Hercules, CA, USA) according to the manufacturer’s instructions. Analyzed cytokines included Eotaxin, G-CSF, GM-CSF, IFN-γ, IL-1α, IL-1β, IL-2, IL-5, IL-6, IL-9, IL-10, IL-12 (p40), IL-12 (p70), IL-17A, KC, MCP-1 (MCAF), RANTES and TNF-α. Cytokines were analyzed using the Bio-Plex 200 system (Bio-Rad Laboratories) and raw data was processed using the Bio-Plex Manager software version 4.1.1 (Bio-Rad Laboratories).

### 2.8. Magnetic Resonance Imaging and ^1^H Magnetic Resonance Spectroscopy

Magnetic Resonance Imaging (MRI) studies were performed in a Bruker Biospec 70/30 scanner using a combination of a linear coil (for transmission) with a head phase array coil (for reception). Animals were anesthetized with isoflurane (3% for induction and 1% for maintenance) and placed in an MRI-adapted stereotaxic holder. Respiration rate and body temperature were continuously monitored during the scans. MRI acquisition protocol included an initial flash sequence (repetition time: 100 ms, echo time: 2.5 ms, field of view: 3 cm, matrix: 128 × 128) to center the Field of View (FOV). For brain anatomical images, we used a T2-weighted axial, coronal and sagittal image (TR = 3700 ms; TE, 31.5 ms; FOV = 17 × 17 mm; Number of Averages = 6; Matrix = 256 × 256; Slice thickness = 0.5 mm). Abdominal images were acquired at axial and coronal planes with a T2 sequence, with and without fat suppression pulse (repetition time 1500 ms; echo time 24 ms; FOV: 35 × 35; Number of Averages = 10; Matriz 256 × 256; slice thickness = 0.8). ^1^H Magnetic Resonance Spectroscopy (^1^H-MRS) were collected at three brain regions: olfactory bulb (8 mm^3^), hippocampus (10.4 mm^3^), and cerebellum (15.6 mm^3^) with the following parameters: TR 2000, TE 20, Number of repetitions 256. Metabolite concentrations were estimated by LCModel (version 6.3-1E) using water as internal reference [34]. Data interpretation was carried out by specialists of the Research Magnetic Resonance service of the Hospital Nacional de Parapléjicos of Toledo.

### 2.9. Tissue Collection

At the end of the study (24-weeks after cell delivery), mice were killed by cervical dislocation and major tissues and organs were harvested. A fraction of the samples was immediately frozen in liquid nitrogen for molecular studies, while the rest was fixed with 4% paraformaldehyde for histological analysis.

### 2.10. RNA Extraction and Quantitative Reverse Transcription PCR

Total RNA was isolated from frozen tissues using the easy-blue total RNA Extraction kit (iNtRON Biotechnology, Inc., Seongnam, Korea). Total RNA (1 µg) was used to synthesize cDNA with the iScript™ cDNA Synthesis Kit (Bio-Rad Laboratories). Quantitative reverse transcription PCR (RT-qPCR) was performed using a ViiA™ 7 Real-Time PCR System (Applied Biosystems, Foster City, CA, USA) and the ViiA™ 7 Software (Applied Biosystems), using the standard instrument protocol. All reactions were performed in a 10-μL reaction mixture volume with 1X forward primer, 1X reverse primer, and 1X SYBR^®^ Green SuperMix Low ROX (BIOLINE GmbH, Luckenwalde, Germany). The relative expression level of human GAPDH gene (TaqMan probe Hs99999905; ThermoFisher Scientific, Madrid, Spain) was normalized to the expression level of mouse GAPDH gene (TaqMan probe Mm99999915; ThermoFisher Scientific).

### 2.11. Histological Analysis

Fixed tissues were embedded in paraffin, sectioned in 5 μm-thick slices and processed for hematoxylin and eosin staining. Histological study of the stained sections was carried out by anatomic pathology specialists of the diagnostic service AnaPath (http://www.anapath.es/index.php (accessed on 19 January 2021)).

### 2.12. Statistical Analysis

Data were expressed as mean±SEM. Data were analyzed using the GraphPad Prism 8 software (GraphPad Software Inc., San Diego, CA, USA). Parametric ANOVA followed by a post hoc test was performed to compare more than two experimental groups. Mixed-model ANOVA and Repeated-measures ANOVA were applied when appropriated. All differences were considered significant at a *p* value < 0.05.

## 3. Results

### 3.1. MSC Culture Expansion in Compliance with Quality Control Standards

We performed an in vitro expansion process of human adipose-derived MSCs that mimics the future clinical trial (Supplemental Figure S1). Thus, to assure that MSC manipulation does not compromise the therapeutic properties and safety of cell products, quality control (QC) standards were tested prior cell transplantation, according to GMP requirements. First, microbial and endotoxin contamination was discarded for each cell dose (Supplemental Table S1). Then, MSC identity and purity was verified at the final cell products (i.e., dose 2 and 4) by examining the expression of cell surface markers and their multilineage differentiation potential. Molecular karyotyping was also carried out to discard genetic alterations (Supplemental Figure S1 and Supplemental Table S1). Results of the QCs indicated that all MSCs met the criteria established for the GMP requirements at the time of cell transplantation.

### 3.2. Intranasal Delivery of MSCs Does Not Affect Mice Welfare and Functional Performance

**Figure 1:**
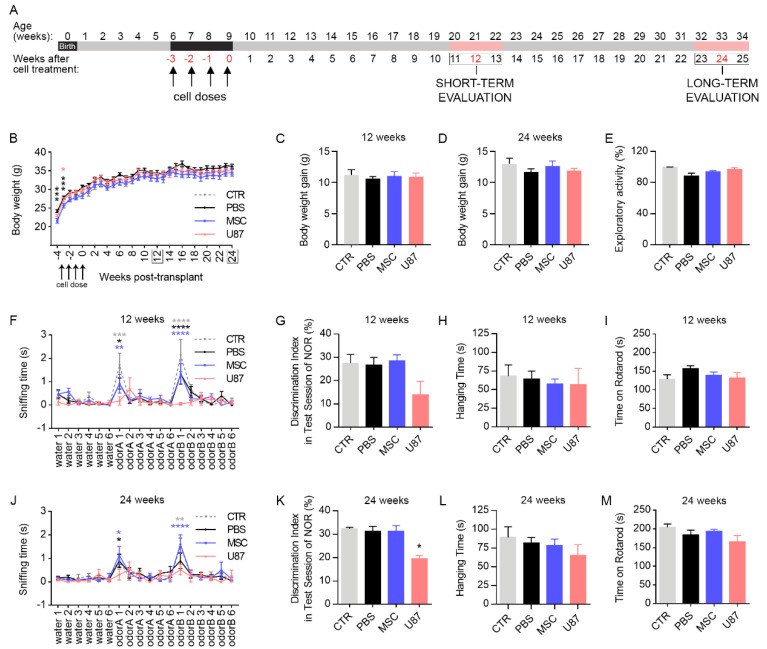
Intranasal delivery of MSCs does not induce changes in mice welfare and functional performance. (**A**) Treated mice received a dose of cells per week during 4 consecutive weeks (5·10^5^ cells/dose). All analyses were conducted between week 11 and 13 for short-term studies (referred as 12 weeks to simplify) and between week 23 and 25 for long-term studies (referred as 24 weeks to simplify). (**B**) Body weight of the animals during the course of the experiment. *n* = 8–20 per group. Color code of the stats correspond to the color of each experimental group (**C**) Body weight gain at the short-term evaluation. *n* = 8–20 per group. (**D**) Body weight gain at the long-term evaluation. *n* = 8–16 per group. (**E**) Exploratory activity of mice prior initiating serial behavioral testing showing no differences between any experimental group. *n* = 4–11 per group. (**F**) Time spent sniffing the stimuli (water, odorA and odorB) in an odor discrimination task at the short-term, showing an impaired olfactory ability in U87 mice. Statistical analysis is performed to detect the change of stimuli (i.e., water 6 vs. odorA 1 and odorA 6 vs. odorB 1) and is indicated with color code corresponding to each experimental group. *n* = 4–13 per group. (**G**) Discrimination index between familiar and novel object (discrimination index = [time exploring the new object-time exploring the familiar object]/[time exploring the familiar object+ time exploring the new object] × 100) in the test session of the Novel Object Recognition (NOR) task at the short-term. *n* = 4–13 per group. (**H**) Wirehang test performance at the short-term. *n* = 4–13 per group. (**I**) Rotarod test performance at the short-term. *n* = 4–13 per group. (**J**) Time spent sniffing the stimuli (water, odorA and odorB) in an odor discrimination task at the long-term, showing an impaired olfactory ability in U87 mice. Statistical analysis is performed to detect the change of stimuli (i.e., water 6 vs. odorA and odorA 6 vs. odorB 1) and is indicated with color code corresponding to each experimental group. *n* = 4–13 per group. (**K**) Discrimination index between familiar and novel object (discrimination index = [time exploring the new object-time exploring the familiar object]/[time exploring the familiar object+ time exploring the new object] × 100) in the test session of the Novel Object Recognition (NOR) task at the long-term. *n* = 12–4 per group. (**L**) Wirehang test performance at the long-term. *n* = 4–12 per group. (**M**) Rotarod test performance at the long-term. *n* = 4–13 per group. Data are represented as mean ± SEM. * *p* < 0.05, ** *p* < 0.01, *** *p* < 0.001, **** *p* < 0.0001 compared to CTR group; Mixed-model ANOVA (**B**), Two-way repeated-measures ANOVA (**F**,**J**). One-way ANOVA (**C**–**E**,**G**–**I**,**K**–**M**).

To evaluate the short- and long-term safety of intranasally delivered MSCs, juvenile athymic nude mice were randomly assigned to four experimental groups: Intact control mice (CTR group), mice receiving intranasal PBS (PBS group), mice receiving intranasal MSCs (MSC group), and mice receiving intranasal positive control cancer cells (U87 group). Cell treatment consisted of a dose of cells per week (5·10^5^ of cells/dose) during 4 consecutive weeks. All analyses were conducted between week 11 and 13 post-transplant for the short-term study (referred as 12 weeks to simplify in the rest of the manuscript), and between week 23 and 25 post-transplant for the long-term study (referred as 24 weeks to simplify in the rest of the manuscript) (Figure 1A).

Mice were monitored after cell transplant to assess animal welfare during the whole study period, using the Welfare Quality^®^ protocol [29]. No significant differences were found in terms of good feeding, housing, health or appropriate behavior, except for mice receiving positive control cancer cells (i.e., U87 mice) (Supplemental Table S2). Over the monitoring period, U87 mice developed frequent and palpable axillary and inguinal masses, as well as subcutaneous swellings in the nose that were rare or absent in the other experimental groups. Differences in body weight between experimental groups were not found after completing cell transplantation (Figure 1B). Similarly, there were no differences in body weight gain in the short- and long-term evaluation (Figure 1C,D).

To further evaluate the safety of intranasal MSC administration, animals were subjected to serial behavioral testing to evaluate olfaction (odor discrimination task), cognition (novel object recognition test), muscle strength (wirehang) and motor coordination (rotarod) on weeks 12 and 24 after cell delivery. Differences in the exploratory activity were firstly discarded between groups to avoid possible confounding results in behavioral testing (Figure 1E). At 12-weeks after cell transplant, odor discrimination task evidenced that MSC and PBS mice habituated to each of the tested odors, as indicated by the reduction in the sniffing time over the 6 sequential presentations of each odor (odorA and odorB; Figure 1F). Then, both MSC and PBS animals were able to detect the novel odor (olfactory dishabituation), as indicated by the increased sniffing time when comparing the sixth sequential presentation of the OdorA and the first sequential presentation of OdorB (Figure 1F). In contrast, U87 mice exhibited and impaired odor discrimination ability. Performance of the novel object recognition test revealed that all experimental groups were able to discriminate the new object at the short-term evaluation (Figure 1G). Similarly, all animals spend the same time in the wirehang and rotarod (Figure 1H,I). At 24-weeks after cell transplant, serial behavioral testing recapitulated the results at the short-term period, except for the novel object recognition test that evidenced a cognitive decline in U87 mice, in addition to the poor odor discrimination ability (Figure 1J–M). These observations demonstrated that intranasal administration of MSCs does not compromise behavioral performances over the monitoring period.

### 3.3. Biochemistry Analysis and Determination of Oxidative Stress-Related Parameters in Blood Samples

**Figure 2:**
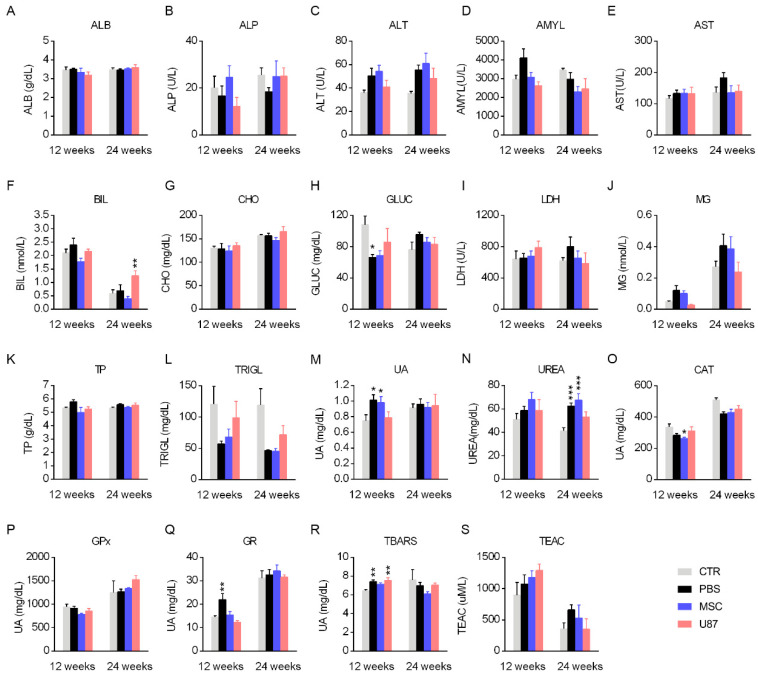
Biochemistry analysis and determination of oxidative stress-related parameters in blood samples. Determination of different biochemical parameters (**A**–**N**) and oxidative stress parameters (**O**–**S**) in overnight fasted mice, 12 and 24 weeks-post cell treatment. ALB, albumin; ALP, alkaline phosphatase; ALT, alanine aminotransferase; AMYL, amilase; AST, aspartate aminotransferase; BIL, bilirubin; CAT, catalase activity; CHO, cholesterol; GLUC, glucose; GPx, glutathione peroxidase; GR, glutathione reductase; LDH, lactate dehydrogenase; Mg, magnesium; TBARS, thiobarbituric acid reactive substances; TEAC, Trolox equivalent antioxidant capacity; TP, total proteins; TRIGL, triglycerides; UA, uric acid. Data are represented as mean ± SEM. *n* = 8–11 per group at 12 weeks-post cell treatment and *n* = 3–11 per group at 24 weeks-post cell treatment. * *p* < 0.05, ** *p* < 0.01, *** *p* < 0.001 compared to CTR group; One-way ANOVA.

Blood samples were collected from mice at 12- and 24-weeks post-transplant to study biochemical and oxidative stress parameters, helping to further detect possible pathologies or functional alterations. Over the monitoring period, biochemistry analyses revealed minimal differences in the plasma metabolite profile of MSC mice, as compared to the CTR group, with punctual parameters altered (i.e., uric acid and urea). Punctual differences were also observed in mice from the PBS and U87 group, as compared to CTR animals (i.e., glucose, uric acid and urea for PBS mice; bilirubin for U87 mice) (Figure 2A–N). At short-term, the parameters of oxidative stress evidenced a reduced catalase activity (CAT) in MSC mice, an increased level of the antioxidant enzyme glutathione reductase (GR) in PBS animals, and an increased level of thiobarbituric acid reactive substances (TBARS), a biomarker of lipid peroxidation, in PBS and U87 mice, when compared to the CTR group (Figure 2O–S). At long-term, the oxidative stress-related parameters did not show differences among groups (Figure 2O–S). Given that only punctual parameters were altered in MSC mice, we suggest that the biochemistry profile and oxidative status of these animals was not influenced by the intranasal administration of MSCs.

### 3.4. Analysis of the Cytokine Profile in Blood Plasma

**Figure 3:**
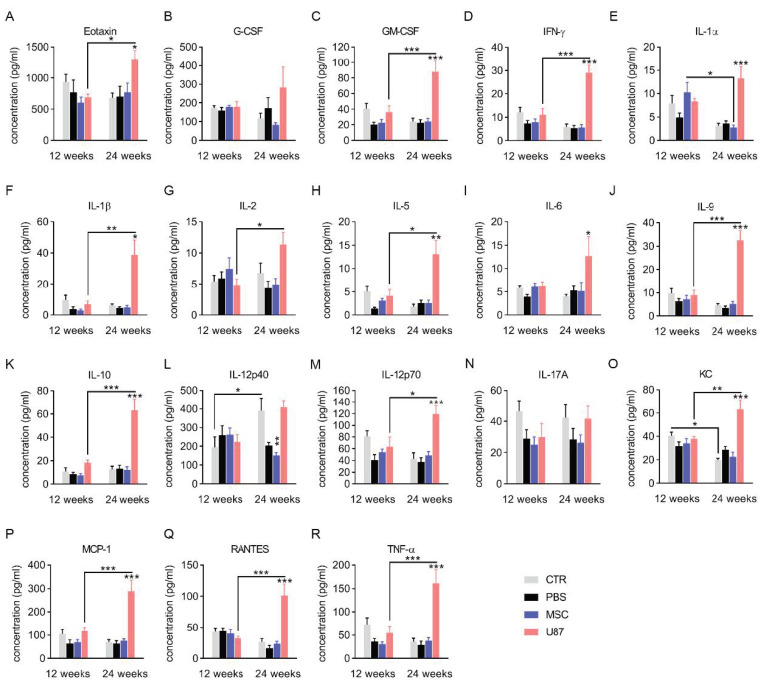
Analysis of inflammatory cytokine in blood plasma. Cytokine profile in the plasma of overnight fasted mice, 12 and 24 weeks-post cell treatment. (**A**–**R**) Bar graphs show the concentration levels of the cytokines. G-CSF, Granulocyte colony-stimulating factor; GM-CSF, Granulocyte Macrophage Colony-Stimulating Factor; IFN-γ, Interferon gamma; IL-1α, Interleukin 1 alpha; IL-1β, Interleukin 1 beta; IL-2, Interleukin 2; IL-5, Interleukin 5; IL-6, Interleukin 6; IL-9, Interleukin 9; IL-10, Interleukin 10; IL-12 (p40), Interleukin 12 subunit p40; IL-12 (p70), Interleukin 12 subunit p70; IL-17A, Interleukin 17A; KC, Keratinocytes-derived chemokine; MCP-1, Monocyte chemoattractant protein-1; TNF-α, Tumor necrosis factor α. Data are represented as mean ± SEM. *n* = 4–10 per group. * *p* < 0.05, ** *p* < 0.01, *** *p* < 0.001 compared to CTR group, unless otherwise indicated; One-way ANOVA.

Plasma samples from mice at 12- and 24-weeks post-transplant were used to study the levels of inflammatory cytokines (Figure 3). The cytokine profile of mice did not reveal evidence of increased susceptibility to inflammation after intranasal administration of PBS or MSCs, as compared to control mice. Interestingly, a decreased level of the pro-inflammatory cytokine IL-12p40 was observed 24-weeks after MSC transplant (Figure 3L). In contrast, mice transplanted with U87 cells exhibited a cytokine storm at the long-term period, with markedly increased levels of multiple inflammatory cytokines, including Eotaxin, GM-CSF, INF-γ, IL-1α, IL-1β, IL-5, IL-6, IL-9, IL-10, IL-12p70, KC, MCP-1, RANTES and TNF- α (Figure 3). While the excessive release of these cytokines suggest a long-term inflammatory reaction in response to transplanted U87 cells, MSC administration did not trigger immune response over time.

### 3.5. Intranasal Administration of MSCs Does Not Induce Anomalies in the Brain and Other Organs In Vivo

**Figure 4:**
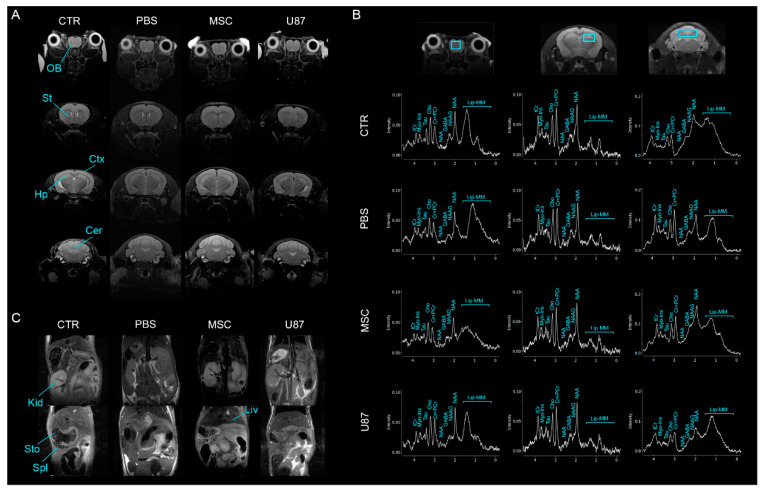
Intranasal administration of MSCs does not induce anomalies in the brain and other main organs in vivo. (**A**) Axial MRI sequence of the brain of a representative MSC animal at the long-term. (**B**) ^1^H-MRS in the olfactory bulb, hippocampus and cerebellum of mice at the long-term. (**C**) Coronal MRI sequence of the abdomen of a representative MSC animal at the long-term. Cr, creatine; Cer, cerebellum; Cho, choline; Ctx, cortex; GABA, g-aminobutyric acid; Hp, hippocampus; Kid, kidney; Lip, Lipid; Liv, liver; MM, macromolecules; Myo-Ins, Myo-inositol; NAA, N-acetylaspartate; NAAG, N-acetylaspartatylglutamate; OB, olfactory bulb; PCr, phosphocreatine; Spl, spleen; St, striatum; Sto, stomach; Tau, taurine; tCr, total creatine. *n* = 3–4 per group.

To investigate whether intranasally delivered MSCs induced anatomical alterations in the brain, we performed MRI across the whole brain at the short- and long-term. Brain scans analyzed by a MRI specialist did not reveal gross anatomical changes among the groups PBS, MSC or U87 over time, as compared to the control group (Figure 4A and Supplemental Figure S2). In order to identify alterations that were undetected by MRI, chemical composition of the brain tissues was evaluated by ^1^H-MRS in the olfactory bulb, the hippocampus and the cerebellum at the long-term (Figure 4B and Supplemental Table S3). Single voxel ^1^H-MRS experiments did not show metabolite changes in the analyzed regions of mice receiving PBS or MSCs, as compared to control animals. However, U87 mice exhibited increased levels of Myo-Inositol (Myo-Ins) and decreased levels of the sum of N-acetylaspartate (NAA) and N-acetylaspartylglutamate (NAAG) in the posterior region of the brain (i.e., cerebellum), as compared to control mice (Supplemental Table S3). Furthermore, evident anatomical alterations in the major abdominal organs were not observed by MRI in any experimental group, at the short- and long-term period (Figure 4C and Supplemental Figure S2).

### 3.6. Long-Term Histological Analysis of the Major Organs Does Not Evidence Lesions after Intranasal Administration of MSCs

**Figure 5:**
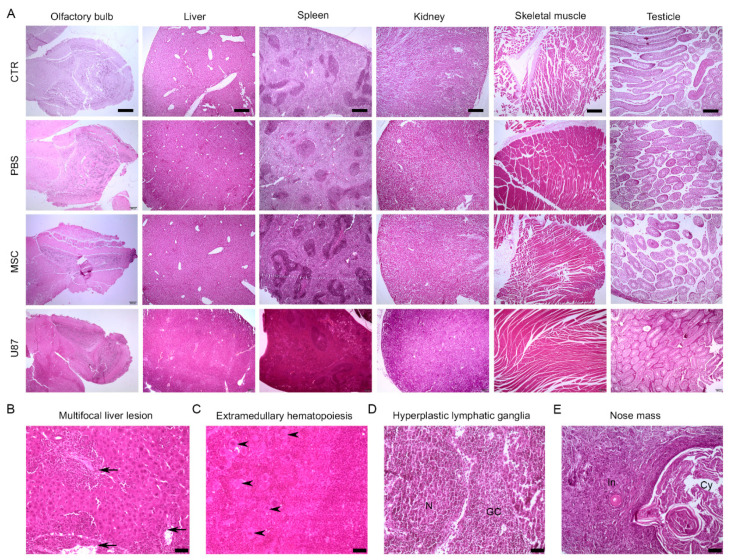
Long-term histological analysis of the major organs does not evidence lesions after intranasal administration of MSCs. Representative images of hematoxylin and eosin staining of major mouse organs for the different experimental groups. (**A**) Comparative histological images of the olfactory bulb, liver, spleen, kidney, skeletal muscle, and testicle. Note that CTR, PBS and MSC animals did not show histological lesions, while U87 mice did. (**B**) Liver tissue from U87 mouse showing cellular infiltration in the periportal area (arrow). (**C**). Spleen tissue from U87 mouse with megakaryocytes (arrowhead) indicating a extramedullary hematopoiesis. (**D**) Atypical axillary mass from U87 mouse with a germinal center (GC) and a nodule (N) that identifies with as hyperplastic lymphatic ganglia. (**E**) Atypical nose cyst (Cy) from U87 mouse with inflammation (In) of the adjacent tissue. Scale bar: (**A**) 400 µm; (**B**–**D**) 50 µm, (**E**) 100 µm. *n* = 3–4 per group.

To investigate whether transplanted cells presented effects in mice at the microscopic level, histological analysis was performed in the major organs (brain, lung, kidney, liver, spleen, skeletal muscle, and testicle) at the long-term. The microscopic examination realized by the anatomic pathology specialists did not reveal tumor formation or abnormalities in the studied organs of MSC mice, supporting MRI results (Figure 5A). The detailed study of the brain, with special attention to the rostral area, did not evidence neoplastic cells after intranasal administration of MSCs. Similarly, there were no histological lesions in any organ of PBS and CTR animals (Figure 5A). In contrast, mice receiving U87 cells presented macroscopic and microscopic lesions (Figure 5A–E). The liver of U87 mice frequently displayed multifocal lesions that were highly infiltrated with inflammatory cells (Figure 5B). Furthermore, extramedullary hematopoiesis occurred in the spleens of U87 animals, observing abundant megakaryocytes (Figure 5C). As previously mentioned, U87 animals presented frequent and palpable axillary and inguinal masses, as well as subcutaneous swellings in the nose over the monitoring period (Figure 5D,E and Supplemental Table S2). The histological analysis of the axillary masses revealed hyperplastic lesions that could be accompanied by inflammatory processes (Figure 5D). The masses in the nose corresponded to follicular cyst with inflammation of the adjacent tissue (Figure 5E).

### 3.7. Biodistribution Suggests a Progressive Disappearance of Transplanted Cells

**Figure 6:**
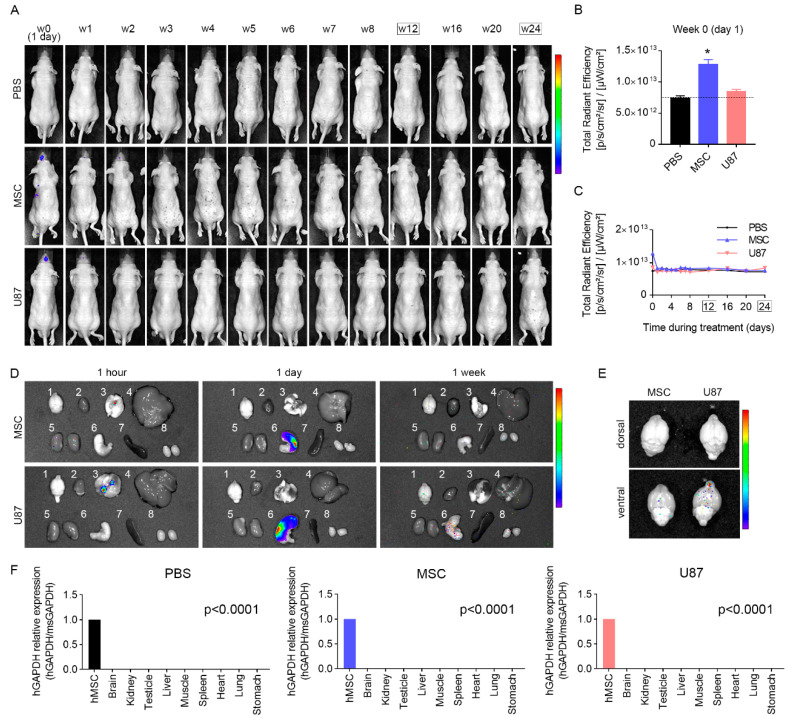
Biodistribution suggests a progressive disappearance of transplanted cells. (**A**) Representative images showing in vivo fluorescence signal in the body of mice at different weeks (w) after cell delivery and until the end of the monitoring period. (**B**) Quantification of the in vivo fluorescence signal in the body at day 1 (week 0) post-transplant. *n* = 4–5 per group. (**C**) Over time quantification of the in vivo fluorescence signal in the body of mice after cell delivery. *n* = 4–5 per group. (**D**) Fluorescence signal in dissected major organs 1 h-, 1 day- and 1 week-post cell delivery. The examined organs were brain (1), heart (2), lungs (3), liver (4), kidneys (5), stomach (6), spleen (7), and testicles (8). (**E**) Fluorescence signal in dissected brains of MSC and U87 mice 1 day-post cell delivery (dorsal and ventral view). (**F**) RT-qPCR quantification of the human GAPDH gene expression in the major organs of PBS, MSC and U87 mice, 24-weeks after cell administration. Expression levels were normalized to the endogenous control mouse GAPDH. *n* = 3 per group. Data are represented as mean ± SEM. * *p* < 0.05 compared to CTR group. One-way ANOVA in B and F. One-way repeated-measures ANOVA in C. Rainbow color scale: red indicates highest fluorescence signal and blue indicates lowest fluorescence signal.

To track the homing of transplanted cells into the different mouse organs, XenoLight DiR dye was used to label cells prior administration. Biodistribution analysis revealed that, within the first 24-h post cell delivery, fluorescence signal was detected in the head, the abdomen and the pectoral region of mice receiving MSC or U87 cells (Figure 6A,B and Supplemental Figure S3). Then, fluorescence in the abdominal and pectoral region tended to gradually decrease, being undetectable at week 1 post-transplant, while fluorescence in the head could be observed until week 4 post-transplant. No signal was found in mice treated with PBS at any time point (Figure 6A,C and Supplemental Figure S3). A cohort of animals was sacrificed to determine the specific organs displaying fluorescence signal in transplanted mice. For this, the brain, heart, lung, liver, kidney, stomach, spleen, and testicles were dissected and examined (Figure 6D). Results revealed that, 1-h post-transplant, fluorescence signal mainly locates in the lung of transplanted mice. The day after cell delivery, the highest fluorescence signal was observed in the stomach. Given that the brain is of particular interest in this study, we examined this tissue separately to avoid a masking effect due to the high fluorescence signal in the stomach. This allowed to observe fluorescence in the brain of transplanted mice 1-day post cell delivery, with the highest signal detected in the olfactory bulbs (Figure 6E). At 1-week post-transplant, specific signal was undetected in any examined organ (Figure 6D).

In order to further examine whether human cells persist in transplanted mice at the end of the monitoring period (24-weeks after transplant), a group of animals was sacrificed and main organs were collected to analyze the expression level of human GAPDH gene. Results indicated that human GAPDH was undetected by RT-qPCR in the brain, lung, kidney, liver, spleen, stomach, heart, skeletal muscle, and testicle of MSC and U87 mice (Figure 6F).

Taken together, these results suggest that human cells graft to specific organs after intranasal delivery, but they tend to gradually disappear, being undetectable in the body of transplanted mice 24 weeks after cell delivery.

## 4. Discussion

The critical challenge for using cell therapy in patients with neurological disorders is how to safely deliver cells into the CNS that efficiently repair the damaged tissue. Intranasal administration has gained recent attention as a non-invasive and feasible method of delivering cells to bypass the BBB and rapidly reach the brain [35]. An increasing number of studies have shown the beneficial effects of intranasal cell delivery for CNS disorders, including Parkinson’s disease, brain tumors, multiple sclerosis, stroke, or Huntington disease, among others [22,23,36,37,38,39,40,41,42,43,44,45,46]. In addition, intranasal cell delivery offers an easy way of performing repeated administration. Previous work from our group demonstrated the therapeutic benefits of repeated dose of intranasally delivered human MSCs to prevent neurological complications of cranial radiation in mice [23]. Irradiated mice exhibited improved motor coordination, cognition and olfaction, 4-weeks after intranasal cell delivery. Moreover, MSC administration was effective in reducing microglia activation, astrogliosis, oxidative damages and neuronal loss in the brain of irradiated mice [23], the major contributors of radiation-induced cognitive toxicity [23,47,48,49,50]. While promising, repeated intranasal cell delivery requires a comprehensive long-term safety study before translation to human clinical trials. The present study was designed to investigate whether multiple doses of intranasally administrated human MSCs in juvenile mice (6-week-old) present adverse effects during adult life (up to 34-week-old). We reported novel data that evidence the long-term safety of repeated dose of GMP-like manufactured human MSCs using the intranasal route.

Following the previous protocol that we used to efficiently reduce the radiation-related brain damages in mice [23], we administrated 5·10^5^ of MSCs once weekly for 4 weeks. During a follow-up period of 24 weeks, no clinical manifestations of toxicity, tumorigenicity or other pathological processes were identified in mice treated with MSCs. Additionally, transplanted cells had no consequences in functional performance, suggesting that intranasal application of MSCs is safe. Similar safety data were previously reported using intramuscular, intravenous and subcutaneous administration of human MSCs in immunosuppressed mice [51,52]. Importantly, human and mouse MSCs have also been transplanted in immunocompetent mice, with no adverse events reported [53,54]. In contrast, animals receiving positive control cancer cells (i.e., U87 cells) exhibited a long-term cytokine storm, which is typically associated with multi-organ failure and death. In line with this observation, U87 mice developed atypical masses in the body and liver lesions over the monitoring period, that were accompanied by impaired olfactory and cognitive function. In addition, the altered brain function found in U87 animals also correlates with the metabolite profiles identified by ^1^H-MRS in the brain. Mice receiving U87 cells exhibited elevated levels of Myo-Ins, which has been associated with poor cognitive performance [55], and reduced levels of NAA + NAAG, which are diminished in several cerebral pathologies, including brain tumors [56]. The unhealthy phenotype observed in mice transplanted with U87 cells highlights the safety of MSC-based therapy. Despite the limited information provided regarding the possible adverse effects of MSCs immediately after transplantation, our study offers a deep investigation of the long-term safety of intranasally administrated human MSCs (see Supporting Information for further details on this limitation).

Several studies have evidenced the beneficial effects of MSCs in a variety of disease, describing mechanisms of action that encompass complex molecular and cellular processes. Among them, the antioxidant properties of MSCs has received considerable attention to explain their cytoprotective and anti-inflammatory effects [57,58]. For instance, the intranasal MSC treatment has been shown to reduce the levels of lysine-4-hydroxynonenal (Lys-4-HNE), a marker of oxidative damage, in the irradiated brain [23]. According with the emerging antioxidant paradigm of MSCs, this favorable environment may account for the protection conferred against radiation-induced neuronal loss and neuroinflammation. Similarly, in the present study, we observed that mice receiving MSCs exhibited a reduced CAT in plasma, suggesting that MSCs may be lowering the oxidative environment, even in the absence of lesion. Importantly, the antioxidant and anti-inflammatory effects of MSCs are also associated with the restoration of cognitive deterioration after radiotherapy [23,59]. It is, therefore, tempting to speculate that intranasally administered MSCs will be also effective in reducing susceptibility to side effects of radiotherapy in humans.

MSCs have a short life after cell transplant [60], as suggested by our biodistribution study. Despite short-lived, we and others have described lasting effects of MSCs, which indicates that MSCs activate other cell types before dying that will carry on the long-term beneficial effects of MSCs. For instance, MSCs can promote the differentiation of regulatory T cells and macrophage via secretion of bioactive factors, conferring anti-inflammatory and immunomodulatory effects [61,62,63]. This is of special relevance for human clinical trials safety, since the possibility of inducing toxic reactions is also reduced. However, further studies should be done to determine whether human MSCs are safe also in patients and to evaluate the fate of these cells in case they survive longer that in our mouse model (see Supporting Information for further details on this limitation). Even so, the paracrine activity of MSCs may be insufficient to alleviate progressive and persistent damages, such as the neurological sequelae experienced by children with brain cancer after radiotherapy [24,25,26,27]. In those cases, a cell therapy that offers the possibility of performing repeated administrations would be of high value. As a non-invasive strategy, a dose of intranasally administrated MSCs could be applied after each session of radiation therapy, with minimal impact for the patient. Here, we assessed doses of cells (~17·10^6^ cells/Kg) in mice that are comparable to those used in clinical studies. For example, an ongoing phase 1/2 clinical trial is assessing the safety and feasibility of intranasally administrated bone marrow-derived allogeneic MSCs to treat perinatal arterial stroke in neonates (≥36 weeks of gestation). In this clinical study, authors administrate 50·10^6^ cells that, for a neonate of 2.8 Kg, correspond to a dose of ~18·10^6^ cells/Kg, similar to our dose. We demonstrated that the intranasal administration of ~17·10^6^ cells/Kg does not cause short- or long-term adverse effects in mice, even when repeated administrations are performed. Our study was carried out in non-irradiated mice to avoid that radiation side effects mask the possible risks of cell therapy. It is important, however, to mention that MSC treatment did not affect mice survival after radiation exposure and it was effective to reduce radiation-induced neuroinflammation, which is associated with cognitive toxicity [23,47,48,49,50] (Supplemental Figure S4). Although long-term safety studies using irradiated mice could provide further information, our neuroprotective strategy has the potential to be a solution for patients suffering neurological sequelae of radiotherapy.

## 5. Conclusions

In conclusion, the present study, in combination with our previous report [23], provides evidence that repeated intranasal administration of MSCs is a safe, simple and effective non-invasive therapy to minimize the side effects of oncological radiotherapy in juvenal and adult mice. These results bring hope for oncological patients that suffer the neurological sequelae of radiotherapy, both children and adults. Furthermore, our data may be taken as a reference point in the design of future clinical trials with MSCs, not only for radiation-related damages but for other CNS disorders.

## Data Availability

The data that support the findings of this study are available from the corresponding author upon reasonable request.

## Supplementary Materials

The following are available online at https://www.mdpi.com/2072-6694/13/5/1169/s1. Supplementary Discussion; Supplementary Material and Methods including Production and QC standards for MSCs; Figure S1: MSC culture expansion in compliance with quality control standards; Figure S2: Intranasal administration of MSCs does not induces anomalies in the brain and other main organs in vivo; Figure S3: Biodistribution of transplanted MSCs; Figure S4: Evaluation of the effects of intranasally delivered MSCs in whole-brain irradiated mice; Table S1: Quality controls of produced MSCs; Table S2: Evaluation of mice welfare; Table S3: Metabolites identify by ^1^H-MRS in the olfactory bulb, hippocampus and cerebellum at the long-term period.
