# Supplementary material for: Preclinical Safety Evaluation of Intranasally Delivered Human Mesenchymal Stem Cells in Juvenile Mice
Source: Cancers (Basel). 2021 Mar 9;13(5):1169. doi: 10.3390/cancers13051169 (PMC7963187; doi:10.3390/cancers13051169)
Supplement: Supplementary file 1 [file cancers-13-01169-s001.pdf]

cancers MDPI

Article

Preclinical safety evaluation of intranasally delivered human mesenchymal stem cells in juvenile mice

Yolanda Aguilera[1], Nuria Mellado-Damas[1], Laura Olmedo-Moreno[1], Víctor López[1], Concepción Panadero-Morón[1], Marina Benito[2], Hugo Guerrero-Cazares[3], Catalina Marquez-Vega[4], Alejandro Martín-Montalvo[1], Vivian Capilla-González[1]*

1 Andalusian Molecular Biology and Regenerative Medicine Centre (CABIMER)-CSIC-US-UPO, Department of Regeneration and Cell Therapy, 41092 Seville, Spain; yolanda.aguilera@cabimer.es (Y.A.); nuria.mellado@cabimer.es (N.M.-D.); laura.olmedo@cabimer.es (L.O.-M.); victorld1991@gmail.com (V.L.); conchipm98@gmail.com (C.P.-M.); alejandro.martinmontalvo@cabimer.es (A.M.-M.)
2 Research Magnetic Resonance Unit, Hospital Nacional de Parapléjicos, 45004 Toledo, Spain; mbenitov@sescam.jccm.es
3 Department of Neurosurgery, Mayo Clinic, Jacksonville, 32224 FL, USA; Guerrero-Cazares.Hugo@mayo.edu
4 Pediatric Oncology Unit, Hospital Virgen del Rocio, 41013 Seville, Spain; catalina.marquez.sspa@juntadeandalucia.es
* Correspondence: vivian.capilla@cabimer.es

SUPPORTING INFORMATION

SUPPLEMENTARY DISCUSSION

It is well known that the culture conditions during the manufacturing process may result in functional heterogeneity of MSCs [1]. For this reason, safety and efficacy studies should be performed using the same batch of cultured MSCs. In a first study, we demonstrated that repeated intranasal applications of human MSCs were effective to prevent neurocognitive decline in whole-brain irradiated mice [2]. However, irradiated mice (transplanted or not with MSCs) did not survive beyond 10 weeks after radiation (Supplemental Figure 4B), likely due to the severity of the radiation-related damages. For this reason, we could not combine efficacy and long-term safety studies using the radiation model, which is a limitation of our research. Consequently, we decided to perform the safety study in non-irradiated mice to avoid that radiation side effects mask the possible risks of cell therapy. Importantly, in order to reduce functional heterogeneity of MSCs, the manufacture process was subjected to quality control (QC) standards of the Good Manufacturing Practice (GMP) requirements.

Our safety study demonstrated that the intranasal delivery of MSCs is safe at 12 and 24 weeks after administration. In addition, animal welfare assessment, that was carried out daily over the whole study period, did not evidence any indicator of compromised health in MSC-transplanted mice at any time point (Supplemental Table 2), suggesting that cell therapy is also safe during the first weeks after transplantation. The lack of adverse events described in this study may be associated with the fact that MSCs are short-lived. Although we cannot fully conclude that cells disappear after week 4 post-transplantation (Figure 6A), we suggest that MSCs had a limited durability after intranasal delivery, based on previous studies. In this regard, a report using immunocompetent mice demonstrated that mouse MSCs have a short survival time (i.e. 1-2 week) after intravenous infusion [3]. Therefore, as proposed by other authors, MSCs might pass on their effects to the host cells before dying via secretion of paracrine factors that will carry on the long-term beneficial effects [4-6]. Even so, we cannot discard the possibility that a small number of MSCs could survive longer after transplantations, being also responsible for the therapeutic effect. In this regard, RT-PCR determinations indicated that if this is the case, the number of cells surviving until week 24 post-transplantation is below the threshold of detection (Figure 6F). Further studies should be performed to determine the survival time of transplanted cells in the donor and to identify potential safety issues shortly after cell therapy (e.g. at 7 weeks after administration).

cancers

SUPPLEMENTARY MATERIAL AND METHODS

Production and QC standards for MSCs

Prior MCS administration, QC standards were performed according to GMP requirements. The QC are briefly described as follow:

Viability: Cell viability was measured by trypan Blue vital dye exclusion test (0,4%, Sigma Aldrich) according to standard procedure assays. Adequacy >90%.

Sterility test: Absence of microbial contamination of final product and culture supernatant was verified by direct inoculation of the sample into test media with Thioglycollate Broth Penase (VWR International Eurolab, S.L, Barcelona, Spain) to detect facultative anaerobes and aerobes, and test media with Tryptic Soya Broth Penase (VWR International Eurolab) for strict aerobes and fungi. Adequacy was reached when all cultures scored negative after a 14-day culture period.

Mycoplasma test: Absence of mycoplasma contamination was verified using the commercial kit Venor GeM (Minerva Biolabs GmbH, Germany). Adequacy: no contamination detected.

Endotoxin test: Endotoxin content was performed by chromogenic Limulus Amebocyte Lysate (LAL)–based kinetic method using Endosafe-PTS system (Charles River Laboratories; Barcelona, Spain), following the manufacturer's instructions. Cartridges with 0.05 - 5.0 EU/mL sensitivity were used in this study. For the endotoxin testing, a test result was considered valid when the percentage of spike recovery was between 50% and 200% with a coefficient of variation less than 25%. Adequacy <5.0 EU/ml.

Karyotyping: Molecular karyotyping performed by Affymetrix Human SNP Array 6.0 and the CytoScan HD Array in the Genome Core Facility of CABIMER.

Flow Cytometry: Product identity and purity were determined by cell surface marker expression by flow cytometry. MSCs were characterized using antibodies against CD13, CD29, CD73, CD90, CD105, CD31, CD34, CD45, and HLA II (BD Biosciences, San José, CA). Data were analyzed using an ACSCalibur Flow Cytometer (BD FACSCalibur cytometry System).

Cell differentiation: MSCs were differentiated into osteoblasts, adipocytes and chondrocytes using lineage specific induction media (Differentiation medium BulletKit™; Lonza, Basel, Switzerland), according to manufacturer´s instructions.

cancers

SUPPLEMENTARY FIGURES

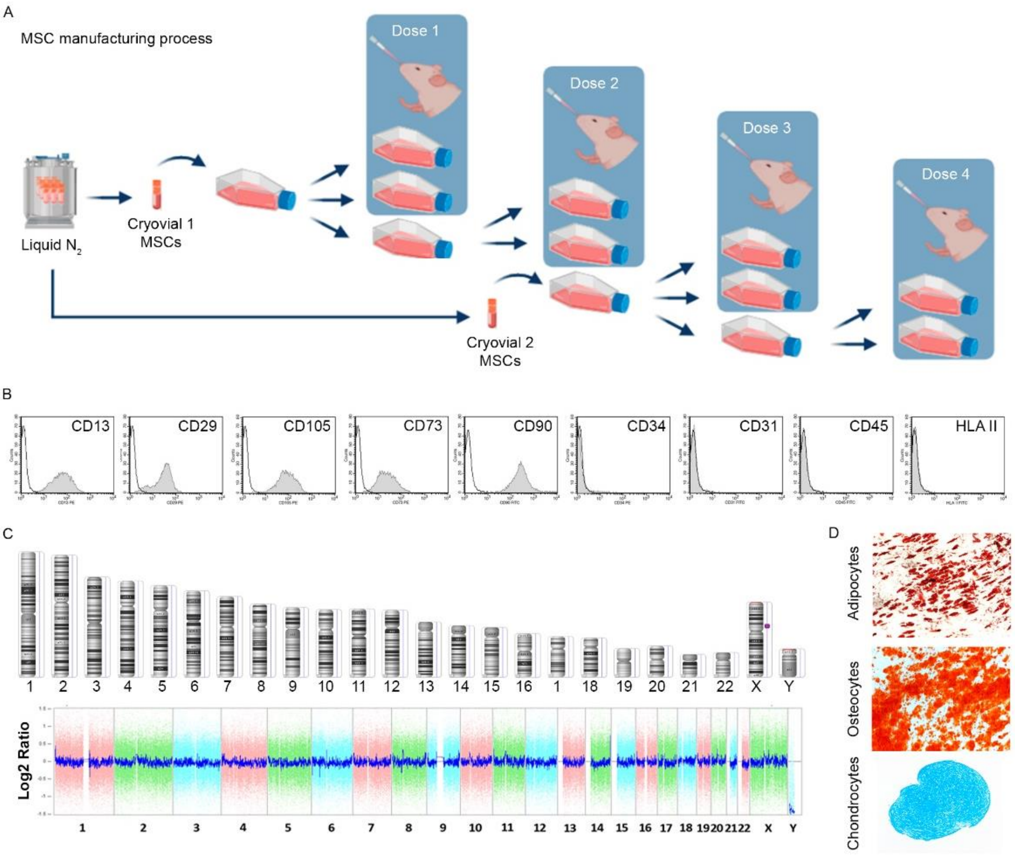

Supplemental Figure S1. MSC culture expansion in compliance with quality control standards. (**A**) Schematic representation of the manufacturing process for the 4 doses of MSCs. QCs were tested for each cell dose prior administration. Image created with BioRender.com (**B**) Representative flow cytometry analysis of cultured MSCs showing positive expression of the MSC-specific markers CD13, CD29, CD73, CD105, and CD90, whereas there was a negative expression for CD31, CD45, CD34, and HLA II. (**C**) Schematic representation of the chromosomes and genome wide SNP array results, showing no genomic alterations in manufactured MSCs. (**D**) Representative microscope images of MSCs differentiated into adipocytes (demonstrated by the presence of lipid droplets stained with Oil Red O), osteocytes (demonstrated by the presence of calcium deposits stained with Alizarin Red), and chondrocytes (demonstrated by the presence of cartilage-specific extracellular matrix components in paraffin-embedded sections of chondrocyte spheroids stained with Alcian blue).

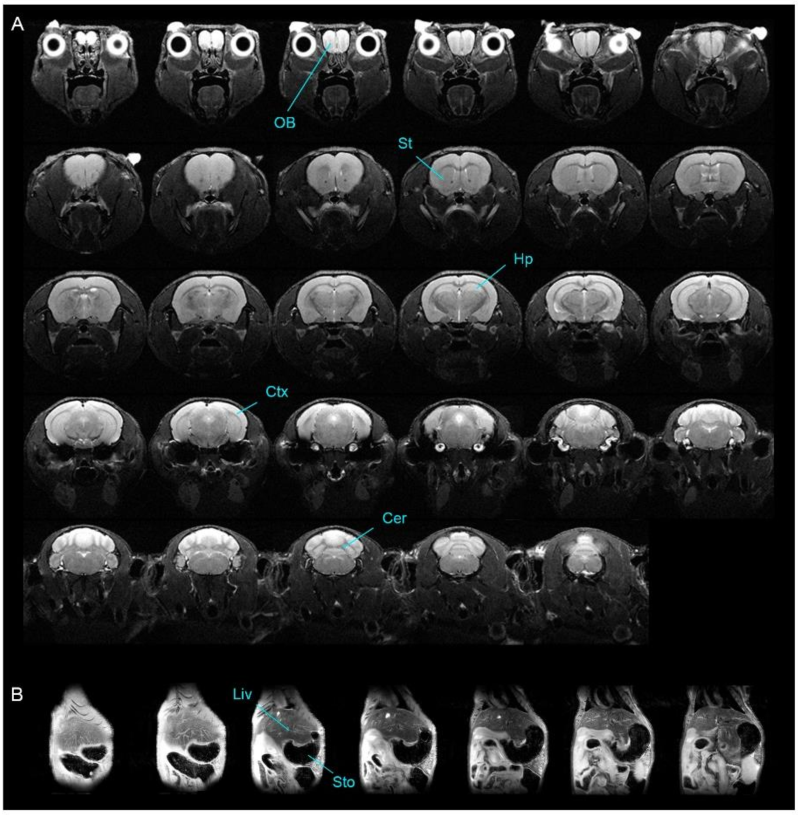

Supplemental Figure S2. Intranasal administration of MSCs does not induces anomalies in the brain and other main organs in vivo. (**A**) Axial MRI sequence of the brain of a representative MSC animal at the short-term. (**B**) Coronal MRI sequence of the abdomen of a representative MSC animal at the short-term. Cer, cerebellum; Ctx, cortex; Hp, hippocampus; Liv, liver; OB, olfactory bulb; St, striatum; Sto, stomach. n = 3-4 per group.

cancers

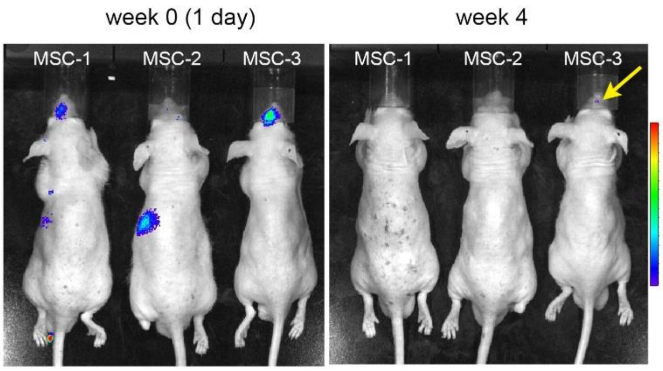

Supplemental Figure S3. Biodistribution of transplanted MSCs. Representative images showing in vivo fluorescence signal in the body of MSC mice the day after cell delivery (i.e. week 0) and 4 weeks after cell transplant (n=5). On week 0, transplanted cells could be detected in the head, the pectoral region, the abdomen (probably the stomach) and the feces of mice transplanted with MSCs. On week 4, low fluorescence signal was restricted to the head (yellow arrow). Rainbow color scale: red indicates highest fluorescence signal and blue indicates lowest fluorescence signal.

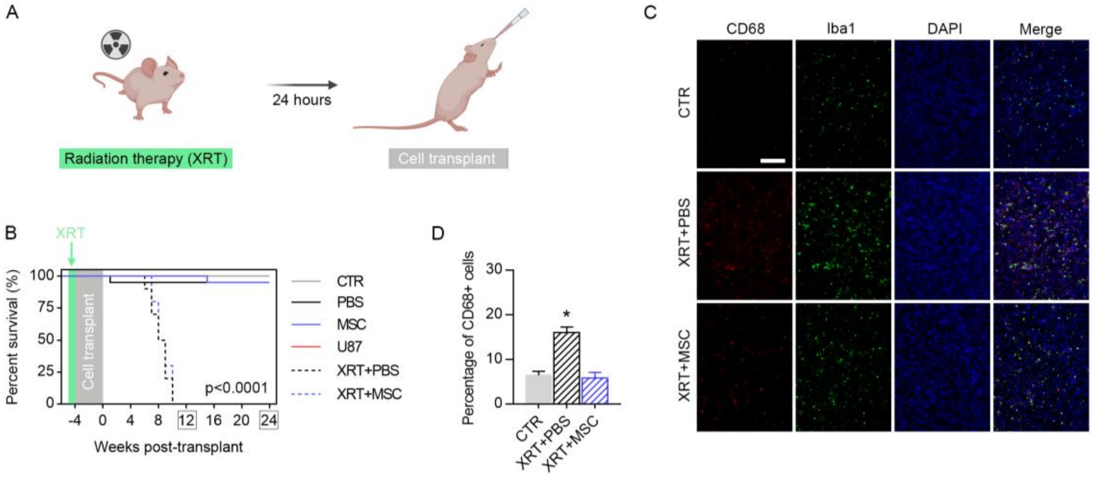

Supplemental Figure S4. Evaluation of the effects of intranasally delivered MSCs in whole-brain irradiated mice. Animals in this study were assigned to six experimental groups: intact control mice (CTR group), mice receiving intranasal PBS (PBS group), mice receiving intranasal MSCs (MSC group), mice receiving intranasal positive control cancer cells (U87 group), mice receiving cranial radiation and intranasal PBS (XRT+PBS group) and mice receiving cranial radiation and intranasal MSC (XRT+MSC group). (**A**) Schema outlining treatments used in the survival study. Mice received a total dose of 10 Gy head-only XRT in 2 fractions (2 x 5 Gy), as previously described [2]. The day after, mice were treated with a weekly dose of MSCs for 4 consecutive weeks ($5 \cdot 10^5$ of cells/dose). Image created with BioRender.com (**B**) Kaplan-Meier curve showing the percentage of survival mice. Survival curves were plotted using the Kaplan-Meier method, which include any animal found dead or euthanized. Note that all whole-brain irradiated mice exhibited shortened survival (i.e., XRT+PBS or XRT+MSC), as compared to non-irradiated mice (i.e., CTR, PBS, MSC, and U87 mice). Importantly, MSC treatment does not affect the time that mice survive after radiation exposure. The red line is not visible because it overlaps with the grey line. Log-rank test. n =9-17 per group. (**C**) Representative immunofluorescence images of the brain of CTR, XRT+PBS and XRT+MSC mice, using CD68 and Iba1 markers, the day after the 4th doses of MSCs (i.e. 4 weeks after radiation). Note that the XRT+MSC group exhibited lower neuroinflammation than XRT+PBS mice, as evidenced by reduced CD68 and Iba1 immunoreactivity. Scale bar, 200 µm. (**D**) Quantification of the number of CD68+ cells shown in C. n=4 per group. Data are represented as mean ± SEM. *p<0.005 compared to CTR group. One-way ANOVA.

SUPPLEMENTARY TABLES

Supplemental Table S1. Quality controls of produced MSCs.

Assay	Specification		Dose 1	Dose 2	Dose 3	Dose 4
Viability	>90%		Meet the criteria	Meet the criteria	Meet the criteria	Meet the criteria
Sterility test	No growth		No growth	No growth	No growth	No growth
Bacterial Endotoxin test	<5EU/mL		<2EU/mL	<2EU/mL	<2EU/mL	<2EU/mL
Mycoplasma	Negative		Negative	Negative	Negative	Negative
Karyotyping	Normal		N/A	Normal	N/A	Normal
Flow cytometry	Positive (>90%) for CD13, CD29, CD73, CD90, CD105	Negative (<5%) for CD31, CD34, CD45, HLA II	N/A	Meet the criteria	N/A	Meet the criteria
Cell differentiation	Confirmed for osteocytes, chondrocytes and adipocytes		N/A	Meet the criteria	N/A	Meet the criteria

cancers

MDPI

Supplemental Table S2. Evaluation of mice welfare.

Welfare principles	Welfare criteria	Mouse parameters	Evaluation			
			CTR	PBS	MSC	U87
Good feeding	Absence of prolonged hunger	Body condition	Normal	Normal	Normal	Normal
		Ability to reach the food hopper	Normal	Normal	Normal	Normal
	Absence of prolonged thirst	Dehydration	Absent	Absent	Absent	Absent
		Ability to reach the water nipple	Normal	Normal	Normal	Normal
Good housing	Comfort around resting	Nest building performance	Normal	Normal	Normal	Normal
	Thermal comfort	Pups outside the nest	n/a	n/a	n/a	n/a
	Ease of movement	Gait/movements	Normal	Normal	Normal	Normal
Good health	Absence of injuries	Lameness	Absent	Absent	Absent	Absent
		Piloerection	n/a	n/a	n/a	n/a
		Hunched position	Absent	Absent	Absent	Absent
		Wounds /excluding bite wounds)	Absent	Absent	Absent	Absent
	Absence of diseases	Urine and feces	Normal	Normal	Normal	Normal
		Coat condition	Normal	Normal	Normal	Normal
		Ocular/nasal discharge	Absent	Absent	Absent	Absent
		Distended abdomen	Absent	Absent	Absent	Absent
		Other deviations (innate/acquired)	Absent	A mouse developed an axillary mass	Absent	Frequent atypical masses
	Absence of pain induced by management procedures	Activity and interactions with environment	Normal	Normal	Normal	Normal
		Facial expression of pain	Absent	Absent	Absent	Absent
Appropriate behavior	Expression of social behaviors	Whisker and/or fur trimming	Normal	Normal	Normal	Normal
		Bite wounds/marks	Normal	Normal	Normal	Normal
		Vocalization/audible fights in cage	Normal	Normal	Normal	Normal
		Blood stains in cage	Absent	Absent	Absent	Absent
	Expression of other behaviors	Circling	Absent	Absent	Absent	Absent
		Jumping against cage wall	Normal	Normal	Normal	Normal
		Bar chewing	Normal	Normal	Normal	Normal
	Good human-animal relationship	Approaching hand in cage	Normal	Normal	Normal	Normal
		Ease of handling when moving mice from dirty to clean cage	Normal	Normal	Normal	Normal
		Urination/defecation during handling	Normal	Normal	Normal	Normal
	Positive emotional states	Rearing	n/a	n/a	n/a	n/a

n=8-20 per group

Supplemental Table S3. Metabolites identify by ^{1}H-MRS in the olfactory bulb, hippocampus and cerebellum at the long-term period.

OLFACTORY BULB					
**Metabolite**	**Mean concentration (Met/Cr+Pcr)**				**ANOVA *p* value**
	**PBS**	**MSC**	**U87**	**CTR**	
Glu	1,02±0.22	0,99±0.04	1,12±0.33	0,90±0.09	0.8833
Ins (Myo-Ins)	0,84±0.22	0,84±0.24	0,89±0.15	0,93±0.23	0.9903
NAA	1,17±0.16	0,87±0.17	1,32±0.06	1,29±0.06	0.1594
Tau	1,56±0.23	1,45±0.3	1,81±0.38	1,51±0.16	0.8106
GPC+PCh	0,23±0.04	0,26±0.01	0,37±0.13	0,28±0.02	0.4938
NAA+NAAG	1,19±0.16	0,87±0.17	1,32±0.06	1,36±0.10	0.1330
Cr+PCr	1,00±0.00	1,00±0.00	1,00±0.00	1,00±0.00	N/A
Glu+Gln	1,21±0.25	1,15±0.11	1,22±0.43	0,95±0.12	0.7755
MM09+Lip09	2,49±1.76	0,85±0.02	1,13±0.59	0,94±0.07	0.5063
**HIPPOCAMPUS**					
**Metabolite**	**Mean concentration (Met/Cr+Pcr)**				**ANOVA *p* value**
	**PBS**	**MSC**	**U87**	**CTR**	
PCr	0,73±0.04	0,56±0.02	0,88±0.12	0,83±0.07	0.1127
GABA	0,36±0.05	0,33±0.02	0,32±0.01	0,34±0.03	0.8681
Glu	0,86±0.05	0,91±0.04	0,93±0.04	1,00±0.06	0.2957
Ins (Myo-Ins)	0,64±0.11	0,38±0.01	0,50±0.02	0,51±0.04	0.1118
NAA	0,82±0.05	0,87±0.03	0,84±0.02	0,86±0.03	0.7698
Tau	0,90±0.31	0,48±0.02	0,53±0.02	0,53±0.06	0.3081
GPC+PCh	0,24±0.01	0,20±0.04	0,23±0.01	0,26±0.02	0.2414
NAA+NAAG	0,84±0.10	0,86±0.10	0,96±0.02	0,98±0.04	0.4506
Glu+Gln	1,19±0.13	1,07±0.06	1,13±0.05	1,16±0.07	0.7831
MM09	1,55±0.98	0,57±0.10	0,62±0.13	0,73±0.08	0.5428
MM09+Lip09	1,90±1.14	0,67±0.09	0,72±0.10	0,81±0.04	0.4608
**CEREBELLUM**					
**Metabolite**	**Mean concentration (Met/Cr+Pcr)**				**ANOVA *p* value**
	**PBS**	**MSC**	**U87**	**CTR**	
PCr	0,83±0.09	0,69±0.02	0,74±0.22	1,00±0.00	0.6807
Glu	0,56±0.02	0,56±0.06	0,55±0.06	0,69±0.06	0.3479
Ins (Myo-Ins)	0,41±0.03	0,45±0.01	0,48±0.04*	0,36±0.06	0.0097
NAA	0,50±0.02	0,56±0.08	0,43±0.06	0,57±0.02	0.1567
Tau	0,37±0.03	0,30±0.01	0,46±0.05	0,26±0.11	0.1619
GPC+PCh	0,18±0.02	0,16±0.02	0,16±0.01	0,20±0.02	0.2282
NAA+NAAG	0,54±0.02	0,67±0.06	0,42±0.05*	0,65±0.04	0.0058
Glu+Gln	0,80±0.09	0,80±0.09	0,73±0.05	0,94±0.12	0.4477

Data are represented as mean ± SEM. n=3-4 per group. *$p<0.05$ compared to CTR group; One-way ANOVA.

References

1. Costa LA; Eiro N; Fraile M, et al. Functional heterogeneity of mesenchymal stem cells from natural niches to culture conditions: implications for further clinical uses. *Cell Mol Life Sci* 2021, *78*, 447-467, doi:10.1007/s00018-020-03600-0.
2. Soria B; Martin-Montalvo A; Aguilera Y, et al. Human Mesenchymal Stem Cells Prevent Neurological Complications of Radiotherapy. *Frontiers in cellular neuroscience* 2019, *13*, 204, doi:10.3389/fncel.2019.00204.
3. Eggenhofer E; Benseler V; Kroemer A, et al. Mesenchymal stem cells are short-lived and do not migrate beyond the lungs after intravenous infusion. *Front Immunol* 2012, *3*, 297, doi:10.3389/fimmu.2012.00297.
4. Azevedo RI; Minskaia E; Fernandes-Platzgummer A, et al. Mesenchymal stromal cells induce regulatory T cells via epigenetic conversion of human conventional CD4 T cells in vitro. *Stem cells* 2020, *38*, 1007-1019, doi:10.1002/stem.3185.
5. Tasso R; Ilengo C; Quarto R, et al. Mesenchymal stem cells induce functionally active T-regulatory lymphocytes in a paracrine fashion and ameliorate experimental autoimmune uveitis. *Investigative ophthalmology & visual science* 2012, *53*, 786-793, doi:10.1167/iovs.11-8211.
6. Vasandan AB; Jahnavi S; Shashank C, et al. Human Mesenchymal stem cells program macrophage plasticity by altering their metabolic status via a PGE2-dependent mechanism. *Scientific reports* 2016, *6*, 38308, doi:10.1038/srep38308.
